# Not just scratching the surface: distinct radular motion patterns in Mollusca

**DOI:** 10.1242/bio.055699

**Published:** 2020-10-21

**Authors:** Carolin Scheel, Stanislav N. Gorb, Matthias Glaubrecht, Wencke Krings

**Affiliations:** 1 Animal Diversity, Center of Natural History (CeNak), Universität Hamburg, Martin-Luther-King-Platz 3, 20146 Hamburg, Germany; 2 Functional Morphology and Biomechanics, Zoological Institute of the Christian-Albrechts-Universität zu Kiel, Am Botanischen Garten 9, 24118 Kiel, Germany

**Keywords:** Feeding, Mollusca, Function, Radula, Radular teeth

## Abstract

The radula is the organ for mechanical food processing and an important autapomorphy of Mollusca. Its chitinous membrane, embedding small radular teeth, is moved by the set of muscles resulting in an interaction with the ingesta, tearing it and collecting loosened particles. Radulae and their teeth can be quite distinct in their morphology and had been of high research interest, but only a few studies have examined the basic functional principles of this organ, the movement and motion during feeding action. Here, the radular motion of 20 representative species, belonging to four major gastropod lineages (Vetigastropoda, Neritimorpha, Caenogastropoda and Heterobranchia) and Polyplacophora, were recorded and classified. Comparisons of the video footage with the scanning electron microscope (SEM) images of the radula resulted in the recognition of functional tooth rows and the correct position of the teeth during feeding. We identified six different types of radular movements, including rotations and bending of the radula itself. In each movement type, different structures act as counter bearings enabling the animals to grab and tear food.

## Abstract (precis)

**Summary:** The radula is the food-processing organ in Mollusca and one key innovation. By employing high resolution video footage we documented distinct motion patterns while feeding and discovered that the animals can use the radula as counter bearings enabling the tearing of large food items.

## INTRODUCTION

Mollusca is the second specious animal phylum (e.g. Chapman, 2009) exhibiting an incredible diversity (e.g. Wanninger and Wollesen, 2019), especially among snails and slugs. This is, to a great extent, enabled by the molluscan key innovation, the radula, the anatomical structure used for mechanical food processing and gathering in most species.

The feeding organ, the buccal mass, is located ventrally to the oral cavity and comprises the odontophoral cartilages, which are surrounded by a chitinous radular membrane (Guralnick and Smith, 1999) with embedded rows of radular teeth that are mineralised in some groups. While feeding, numerous muscles stretch and pull the membrane over the odontophore (e.g. Katsuno and Sasaki, 2008) bringing the teeth into a feeding position, which allows an interaction with the ingesta. In some taxa, the radula is subsequently pulled to a hard chitinous structure at the ventral and anterior part of the mouth opening, the jaw (e.g. Märkel, 1957; Montroni et al., 2019; Krings et al., 2019a). This structure can be found in all molluscan taxa (Vortsepneva et al., 2013, 2014) except for Polyplacophora and – of course – Bivalvia, but is usually not a prominent structure involved in the feeding process. The loosening, collecting and tearing of ingesta leads to tooth wear (e.g. Runham, 1962; Runham and Thornton, 1967; Mackenstedt and Märkel, 1987; Shaw et al., 2010), but through a continuous growth of the radular ribbon from posterior to anterior, tooth rows constantly break loose and are replaced (e.g. Runham, 1962; Mackenstedt and Märkel, 1987). Radulae and radular teeth show highly distinct morphologies throughout the molluscan phylum and in order to gain the upper hand over the overflowing diversity, radulae have been previously categorised into five to eight basic types (e.g. docoglossan, rhipidoglossan, taenioglossan, stenoglossan, toxoglossan, ‘isodont’) based on the quantities and arrangements of certain tooth types (central, lateral and marginal tooth types; see Figs 4–7) per row (e.g. Gray, 1853; Hyman, 1967; Steneck and Watling, 1982; Haszprunar and Götting, 2007). These types do not always and consistently reflect phylogeny due to convergences (Haszprunar et al., 2011) and also ecological adaptations have a strong influence (e.g. Steneck and Watling, 1982; Hawkins et al., 1989), but overall morphology of the radula and its teeth is a valuable character in taxonomy and systematics (e.g. Anistratenko et al., 2013).

Overall the structure of the buccal mass and the anterior part of the digestive system has been of high research interest throughout the decades (e.g. Geddes, 1879; Plate, 1897; Amaudrut, 1898; Herrick, 1906; Crofts, 1929; Carriker, 1943; Starmühlner, 1952; Hubendick, 1956; Graham, 1964, 1973; Fretter, 1965; Carriker et al., 1974; Morris and Hickman, 1981; Guralnick and Smith, 1999; Katsuno and Sasaki, 2008; Mikhlina et al., 2018; Krings et al., 2019a), but most of the studies mentioned above, with a few exceptions, focus on single or few species, due to the overflowing molluscan biodiversity. Interestingly, although much is known about this feeding organ, basic principles like the radular movement during feeding are not known for the majority of molluscs. Usually, the feeding motion described in textbooks is the feeding pattern documented for Heterobranchia, which can be described as a licking movement involving the protruding of the radula enabling the teeth to loosen the ingesta (for details see, e.g. Mackenstedt and Märkel, 2001; Neustadter et al., 2002a,b; Montroni et al., 2019; Kehl et al., 2019; Krings et al., 2019a). However, studies on the musculature of the buccal mass (e.g. Graham, 1973; Fretter and Graham, 1976; Crampton, 1977; Morse, 1984; Simone, 2005, 2011; Evans et al., 2009; Golding et al., 2009; Balog et al., 2012) and the analysis of feeding tracks (e.g. Ankel, 1936, 1938; Eigenbrodt, 1941; Hickman and Morris, 1985; Janssen and Triebskorn, 1987; Mackenstedt and Märkel, 2001) demonstrate that the motion apparatus is highly diverse and complex. Traditionally the radula was compared to a flat ribbon or a ‘belt-and-pulley’ model (Ankel, 1938; Märkel, 1964; Solem, 1974; Hickman, 1980), but this view has subsequently changed. The radular movement is quite complex in all three dimensions: in 1981 Morris and Hickman addressed this topic by artificially protruding gastropod radulae from dead specimens. In this context, it must be highlighted that due to bending and stretching of the membrane, teeth of many taxa do not act as individual and independent structures on the ingesta, but rely on each other, transmitting and distributing forces and stresses from one tooth to another during feeding (Solem, 1972; Morris and Hickman, 1981; Hickman, 1984; Padilla, 2003; Herrera et al., 2015; Montroni et al., 2019; Krings et al., 2019b). Hence, the interaction of individual teeth in the radula is quite complex (see Padilla, 2003) and in order to get a hold on the morphological diversity of radulae and connect this with possible functional adaptations, the detailed movement of the radula and the interaction of the teeth with the ingesta/substrate, including orientation, rotations or twists, must be documented. There are few studies on the diversity of radular motion patterns (Ankel, 1938; Eigenbrodt, 1941; Wägele, 1983; Hawkins et al., 1989), but nowadays with high resolution video footage taken in combination with the excellent optics of modern light microscopes, we might be able to build hypotheses on the evolution of feeding motions against the background of available phylogenetic trees. Here, this was done for 19 gastropod species belonging to four major lineages (Vetigastropoda, Neritimorpha, Caenogastropoda and Heterobranchia) and one Polyplacophora.

## RESULTS

### Radular types and tooth formulas

Radulae of analysed specimens (Figs 4–7 and Supplementary data) were assigned to four radular types: docoglossan, rhipidoglossan, taenioglossan and isodont. Radular formulas were generated [with R=the central tooth (CT), flanked to the sides by a certain number of lateral teeth (LTs) or dominant lateral tooth (DT), followed by the N of marginal teeth (MTs)].

The docoglossan type was found in the Polyplacophora *Lepidochitona cinerea* (Fig. 4B,C and Supplementary data). A small CT is flanked to each side by two LTs, followed by one DT and one MT (1+DT+1+R+1+DT+1). Radulae of the Vetigastropoda *Rochia conus* (Fig. 5B and Supplementary data) and *Astralium calcar* (Fig. 5C and Supplementary data), the Neritimorpha *Clithon corona* (Fig. 4F and Supplementary data) and *Vittina turrita* (Fig. 4E and Supplementary data) were assigned to the rhipidoglossan type. Radulae display one CT, flanked by five LTs and numerous MTs (∞+5+R+5+∞). Many of the species studied here show a taenioglossan radula, one character typical in Caenogastropoda: *Stenomelania torulosa* (Supplementary data), *Stenomelania macilenta*, *Thiara cancellata* (Fig. 5D and Supplementary data), *Brotia herculea* (Fig. 5F and Supplementary data), *Faunus ater* (Supplementary data), *Marisa cornuarietis* (Fig. 6B,C and Supplementary data), *Monetaria annulus* (Fig. 5H and Supplementary data), *Littorina littorea* (Fig. 5E and Supplementary data) and *Taia naticoides* (Fig. 5G and Supplementary data). This type contains seven teeth per row, one CT, flanked to each side by one LT and two MTs (2+1+R+1+2). The isodont type was found in Heterobranchia *Cornu aspersum* (Supplementary data), *Trochulus villosulus* (Fig. 7B and Supplementary data), *Lymnaea stagnalis* (Fig. 7C–E and Supplementary data), *Stenophysa marmorata* (Fig. 6E–F and Supplementary data), *Planorbarius corneus* and *Planorbella duryi* (Fig. 7D and Supplementary data). One small CT is flanked by numerous LTs and MTs, all of these teeth are quite similar and symmetrical (∞+∞+R+∞+∞).

### Radular motion patterns

The radular motions of the 20 analysed species were categorised, resulting in six radular motion types, which were split into three phases (Figs 4–7). The first phase is characterised by the protruding of the radula. In the second phase, food particles are sheared, crushed and collected. The last phase comprises the retraction of the organ into the oral cavity. As already described by Hickman (1984), the teeth of different transverse/ontogenetic rows act in concert and assemble to functional rows (functional rows are highlighted in grey in Figs 4–6 and Supplementary data; in Fig. 4C teeth of one transverse/ontogenetic row are highlighted with black boxes).

Radular motion pattern I was observed in *L. cinereal* (Polyplacophora). In phase I, the radula is protruded to the mouth opening, simultaneously the LTs are unfolded to the sides like a fan (Fig. 4A). This curved flexion is reinforced in phase II and reversed in phase III when the radula is retracted. The rotation of the teeth, especially of the DTs, allows them to grab larger food items (counter bearing I).

The second pattern (II) was found in *C. corona* and *V. turrita* (Neritimorpha) and is characterised by a bending or folding of the radula running between the tooth rows (Fig. 4D). In phase I the anterior radular part (Fig. 4D, purple colour) is protruded while being moved in anterior–dorsal direction, followed by a curling or rolling motion in posterior–ventral direction, enabling the shearing of ingesta during phase II. In phase III the anterior part is first retracted into the mouth opening, but since the radula is still bent this retraction allows the posterior part (Fig. 4D, pink colour) to gather the particles and transport them into the oral tube (counter bearing II). All teeth run almost parallel to each other during all phases.

The third pattern (III) was observed in Caenogastropod species, such as *S. macilenta*, *S. torulosa*, *T. cancellata*, *B. herculea*, *F. ater*, *M. annulus*, *L. littorea* and *T. naticoides*, and Vetigastropod species, such as *R. conus* and *A. calcar*, (Fig. 5A) and is similar to motion pattern II (Fig. 4D). However, here, a sharp bending running along the tooth rows was observed, and additionally, phase II is characterised by an abrupt tearing motion in posterior direction. Subsequently the bent anterior and posterior radular parts converge, allowing the tearing and grabbing of large food items (counter bearing III). This motion pattern, in contrast to pattern II, is also characterised by a presumably lateral tension of the radular membrane resulting in MTs sticking out to the sides.

Motion pattern IV was observed in *M. cornuarietis* (Caenogastropoda) and is, like pattern III, characterised by a sharp bent radula (counter bearing IV). However, in the phase II of the pattern IV, the radula additionally shakes and vibrates in the lateral direction (Fig. 6A).

The fifth motion pattern (V) was found in the Heterobranch species, such as *C. aspersum*, *T. villosulus*, *L. stagnalis*, *P. corneus* and *P. duryi* (Fig. 7A). After the protruding of the radula in phase I, the lateral edges of the radula are pulled caudally until the whole structure is formed into a concave shape like a spoon. While moving into anterior direction, the ingesta is loosened and collected, until the organ reaches its counter bearing, the jaw. Both structures together enable the ability to pull and tear large food items (counter bearing V).

Motion pattern VI was only detected in Heterobranch *S. marmorata* (Fig. 6D) and is quite unique. Here, the radula is bent along the CTs in anterior–posterior direction; the inner part of the radula is pulled caudally until a u-shape is achieved. Both lateral wings are flapped, gripping and holding the ingesta (counter bearing VI) until the organ with the food is retracted into the oral cavity.

## DISCUSSION

We were able to detect six distinct radular motion patterns by examining only 20 molluscan species. However, as already mentioned above, the musculature of the buccal mass is quite diverse between molluscan taxa (e.g. Graham, 1973; Fretter and Graham, 1976; Crampton, 1977; Morse, 1984; Simone, 2005, 2011; Evans et al., 2009; Golding et al., 2009; Balog et al., 2012) and feeding tracks demonstrate the complex motion and tooth–ingesta interaction (e.g. Ankel, 1936, 1938; Eigenbrodt, 1941; Hickman and Morris, 1985; Janssen and Triebskorn, 1987; Mackenstedt and Märkel, 2001). Since Mollusca represent the second specious animal phylum (e.g. Chapman, 2009), we expect to find more radular motion pattern by increasing the quantity of species in future studies.

Within the major gastropod taxa, we found similar radular movements. For example, motion pattern I was only observed in Polyplacophora and pattern II in Neritimorpha species: both types are presumably restricted to these lineages. Motion pattern III was observed in Caenogastropods (see also Eigenbrodt, 1941; Wägele, 1983), with modifications involving additional vibrational movements in the lateral directions (motion pattern IV), and Vetigastropods. Hence, the bending of the radula could be plesiomorphic, but more species need to be analysed to build solid hypotheses on the evolution of this motion patterns. Within Heterobranchia, we observed motion pattern V in most species (also described in e.g. in Eigenbrodt, 1941; Wägele, 1983; Mackenstedt and Märkel, 2001; Neustadter et al., 2002a,b; Montroni et al., 2019; Kehl et al., 2019; Krings et al., 2019a), only *S. marmorata* was distinct (motion pattern VI; see also Eigenbrodt, 1941). We would, hence, propose that the licking movement is plesiomorphic in Heterobranchia and was modified within the Physidae.

In all motion patterns, structures were found that act as counter bearings enabling the animals to grab and tear larger pieces of ingesta (Figs 4–7). DTs interact and function as graspers (type I). The bending along the radular tooth rows enables the teeth to clamp items (pattern II–IV). The interplay between the anterior part of the radula and the jaw allows pulling and tearing (pattern V). The radular bending along the anterior–posterior axis allows a flapping and gripping (pattern VI). Hence, it can be concluded that various types of counter bearings for the processing of large food items have convergently evolved in Mollusca.

The distinct motion patterns are presumably the result of the different structural organisation of the radular and buccal mass muscles (e.g. Graham, 1973; Fretter and Graham, 1976; Crampton, 1977; Morse, 1984; Simone, 2005, 2011; Evans et al., 2009; Golding et al., 2009; Balog et al., 2012) and the radular supporting structures, which are of different shape, quantity, volume (see e.g. Mackenstedt and Märkel, 2001; Katsuno and Sasaki, 2008; Golding et al., 2009) and probably bending capacity. The precise interaction of these structures enables the functioning and feeding performance. However, to understand the role of all structures involved in feeding, x-ray recordings of living specimens would be necessary. This approach together with high-resolution video footage of the radular motion could result in a 3D visualisation and animation of the food-gathering process leading to a profound understanding of radular function.

To conclude, our video footage allowed us to document the orientation and position of radular teeth (Figs 4–7 and Supplementary data; see also Eigenbrodt, 1941; Wägele, 1983) which shows no congruence with the traditional malacological depictions except für Heterobranchia, and the assembly of teeth from different transverse/ontogenetic rows to functional rows (functional rows are highlighted in grey in Figs 4–7 and Supplementary data, one transverse/ontogenetic row is highlighted with black in Fig. 4C; see also Hickman, 1984).

## MATERIALS AND METHODS

The analysed gastropod species (*N*=19, see Table 1) were chosen, because they were easy to acquire in custom aquatic retail stores or in the field (on the North Sea, at Husum, Germany in autumn 2019), and because they cover most major lineages (Caenogastropoda, Neritimorpha, Vetigastropoda and Heterobranchia). Additionally, one Polyplacophora species was examined (Table 1). Identification was based on the morphospecies concept, in this case on conchological data, assignment to taxa based on relevant and pertinent literature, and hypotheses on the evolution of radular motion patterns on the phylogenies of Aktipis et al. (2008) and, in case of the Heterobranchia, Jörger et al. (2010).

**Table 1.**
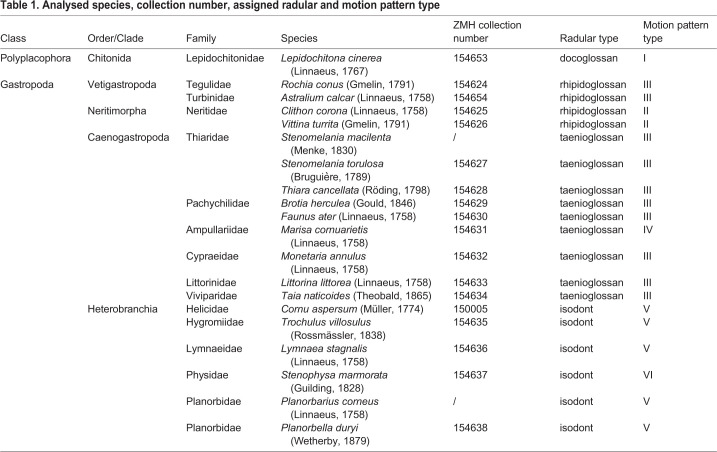
**Analysed species, collection number, assigned radular and motion pattern type**

**Fig. 1.**
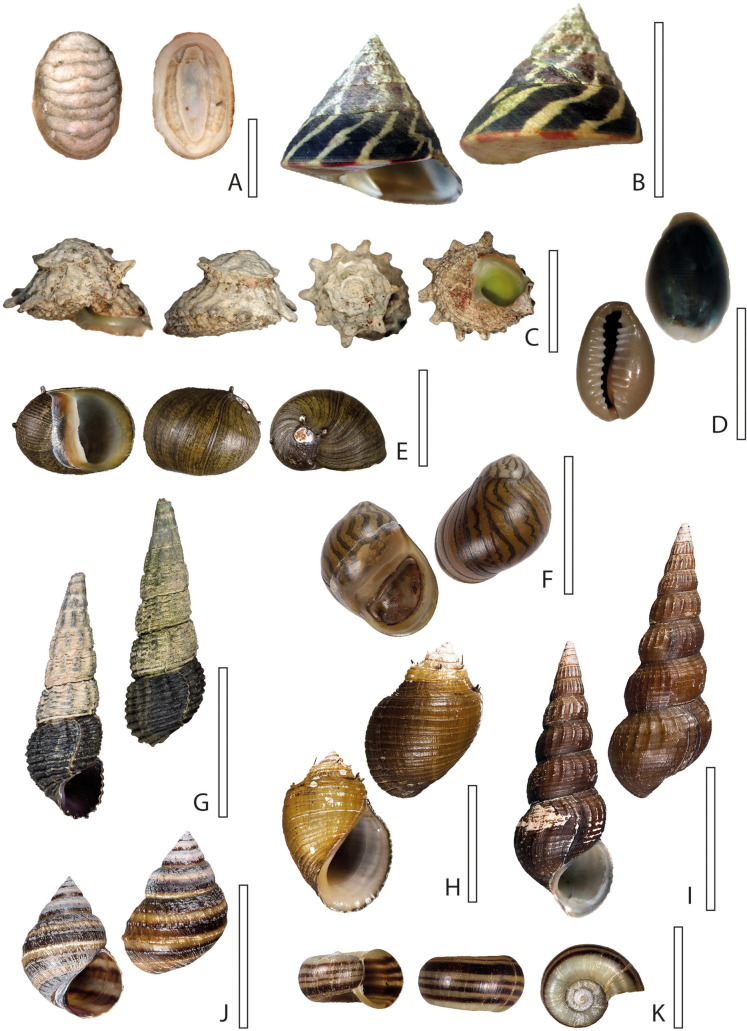
**Shells of analysed species.** (A) *Lepidochitona cinerea*, (B) *Rochia conus*, (C) *Astralium calcar*, (D) *Monetaria annulus*, (E) *Clithon corona*, (F) *Vittina turrita*, (G) *Stenomelania torulosa*, (H) *Thiara cancellata*, (I) *Brotia herculea*, (J) *Taia naticoides* and (K) *Marisa cornuarietis*. Scale bars: A, 5 mm; B, 60 mm; C, 25 mm; D, 20 mm; E, 12 mm; F,G,I,J, 30 mm; H,K,15 mm.

**Fig. 2.**
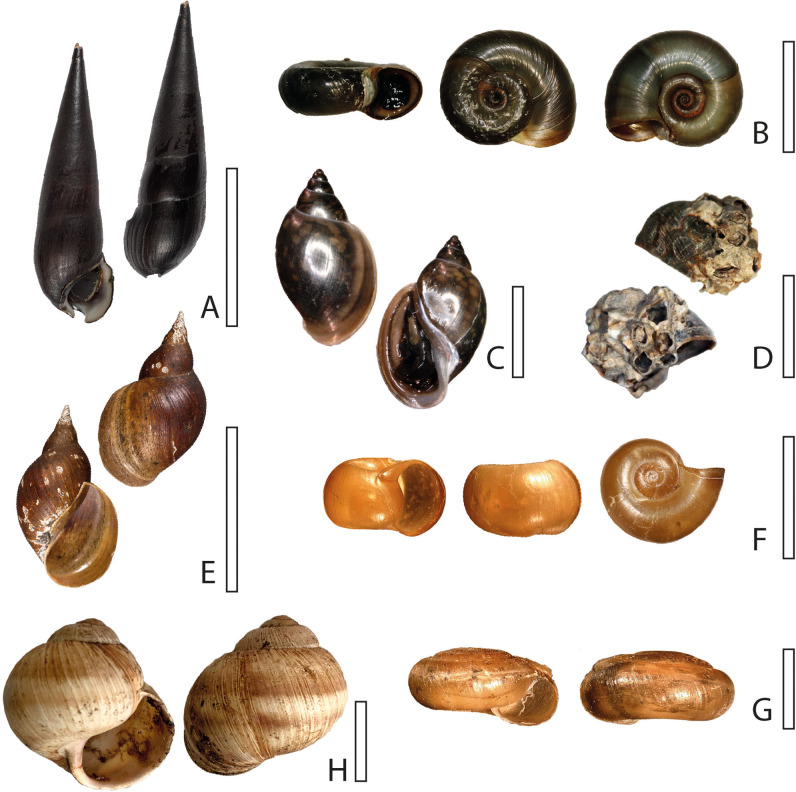
**Shells of analysed species.** (A) *Faunus ater*, (B) *Planorbarius corneus*, (C) *Stenophysa marmorata*, (D) *Littorina littorea*, (E) *Lymnaea stagnalis*, (F) *Planorbella duryi*, (G) *Trochulus villosulus*, (H) *Cornu aspersum*. Scale bars: A,E, 30 mm; B, 35 mm; C, 4 mm; D, 12 m; F, 10 mm; G, 2 mm; H, 15 mm.

**Fig. 3.**
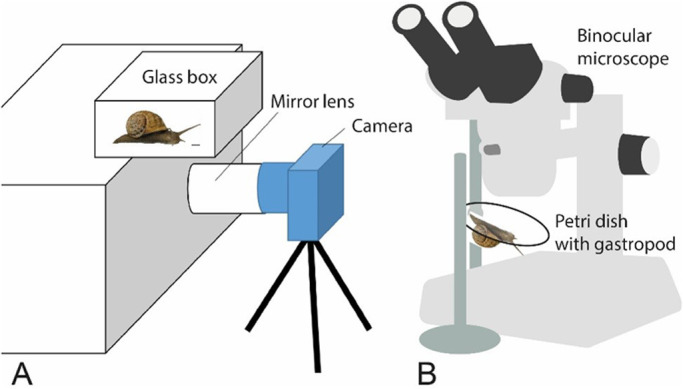
**Schematic illustration of the experimental setup.** (A) Video footage from below with mirror lens and camera. (B) Video footage on small specimens from above with a binocular microscope.

**Fig. 4.**
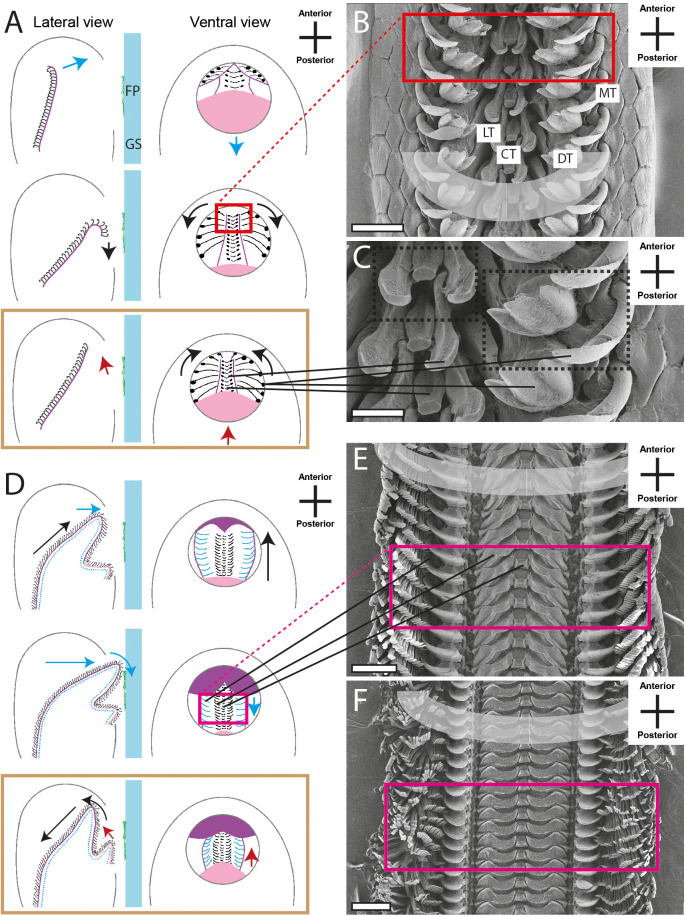
**Schematic illustrations of radular motion patterns I (A) and II (D) in lateral and ventral view as seen through the glass surface.** Blue arrow, motion in ventral direction; red arrow, motion in dorsal direction; black arrow, motion in horizontal/lateral direction; colored frames (red, pink) and black lines link these illustrations with SEM images of teeth (B,C, *Lepidochitona cinerea*; E, *Vittina turrita*; F, *Clithon corona*). Brown frames emphasise the phase where two structures act as counter bearings. CT, central tooth; DT, dominant lateral tooth; FP, food paste; GS, glass surface; LT, lateral tooth; MT, marginal tooth. Scale bars: B,100 μm; C, 30 μm; E,F, 200 μm.

**Fig. 5.**
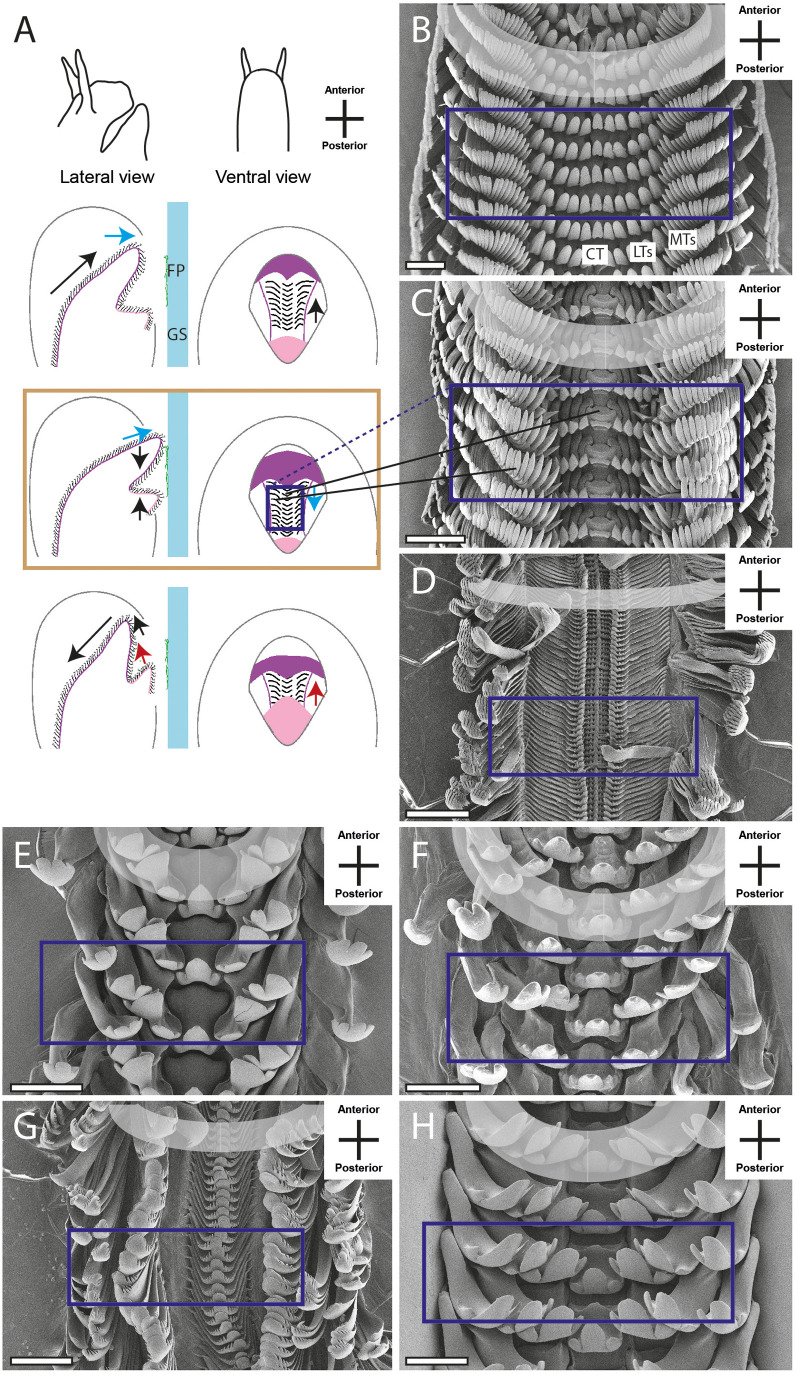
**Schematic illustrations of radular motion pattern III (A) in lateral and ventral view as seen through the glass surface.** Blue arrow, motion in ventral direction; red arrow, motion in dorsal direction; black arrow, motion in horizontal/lateral direction; colored frame (blue) and black lines link these illustrations with SEM images of teeth (B, *Rochia conus*; C, *Astralium calcar*; D, *Thiara cancellata*; E, *Littorina littorea*; F, *Brotia herculean*; G, *Taia naticoides*; H, *Monetaria annulus*). Brown frame emphasises the phase where two structures act as counter bearings. Scale bars: B, 400 μm; C,D,F,G, 200 μm; E, 80 μm; H, 100 μm.

**Fig. 6.**
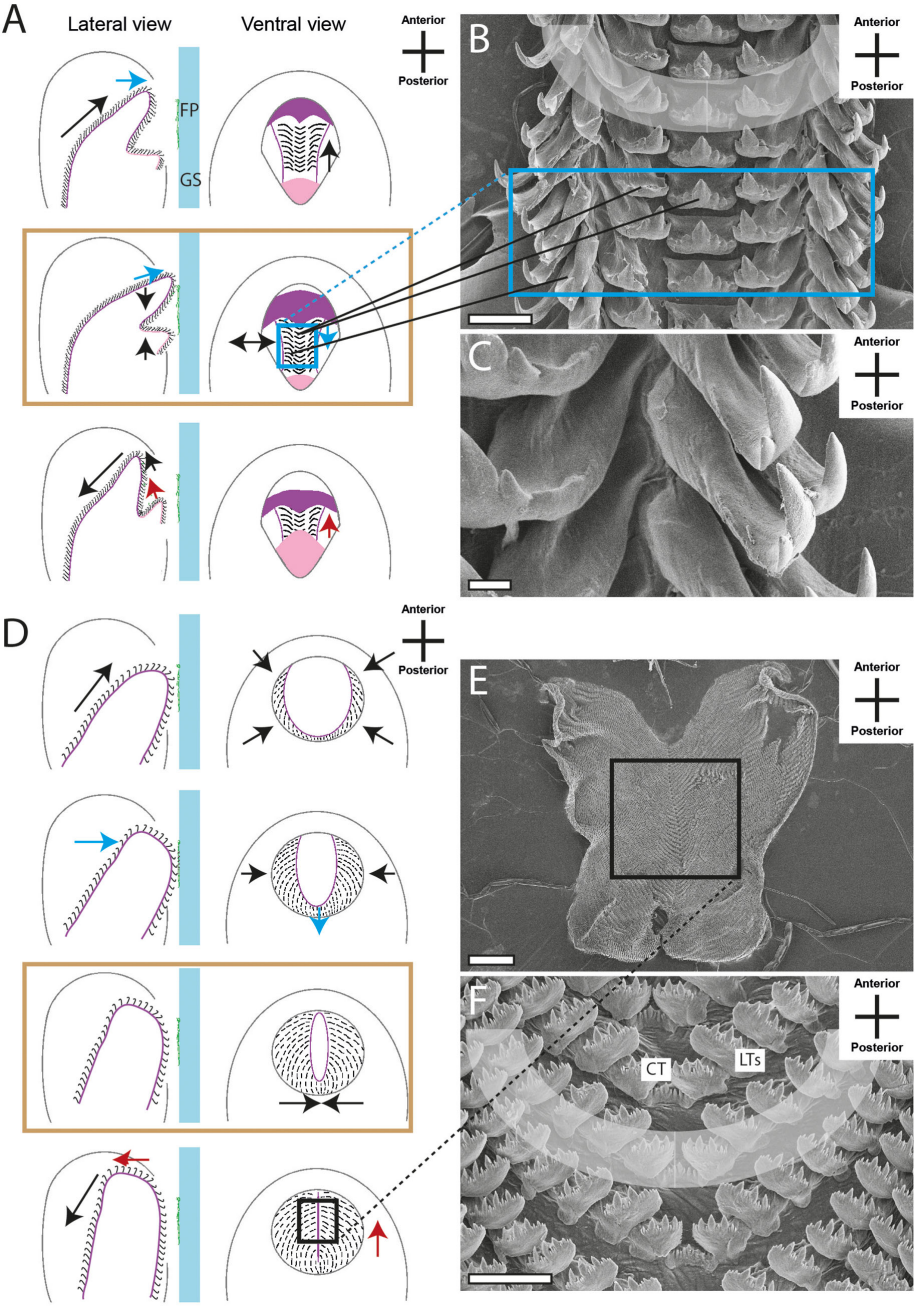
**Schematic illustrations of radular motion patterns IV (A) and VI (D) in lateral and ventral view as seen through the glass surface.** Blue arrow, motion in ventral direction; red arrow, motion in dorsal direction; black arrow, motion in horizontal/lateral direction; colored frame (blue, black) and black lines link these illustrations with SEM images of teeth (B,C, *Marisa cornuarietis*; E,F, *Stenophysa marmorata*). Brown frames emphasise the phase where two structures act as counter bearings. Scale bars: B,E, 200 μm; C, 40 μm; D, 20 μm.

**Fig. 7.**
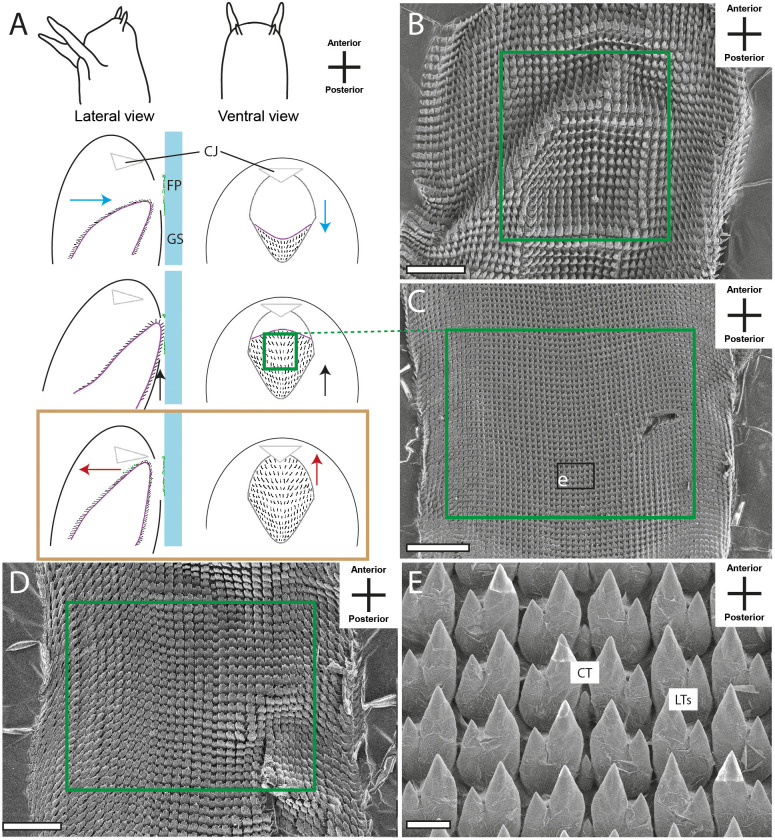
**Schematic illustrations of radular motion pattern V (A) in lateral and ventral view as seen through the glass surface.** Blue arrow, motion in ventral direction; red arrow, motion in dorsal direction; black arrow, motion in horizontal/lateral direction; colored frame (blue, black) and black lines link these illustrations with SEM images of teeth (B, *Trochulus villosulus*: C,E, *Lymnaea stagnalis*; D, *Planorbella duryi*). Brown frame emphasises the phase where two structures act as counter bearings. CJ, chitinous jaw; scale bars: B,D, 100 μm; C, 400 μm; E, 20 μm.

In order to obtain detailed information about the radular movement and motions, we developed the following experimental set-up (Fig. 3A): since some species avoid feeding on a vertical surface presumably due to their weight or size, video footage was obtained by filming a small glass tank from underneath with a Nikon D810 (Nikon Corporation, Tokyo, Japan), a 36.3-megapixel full-frame digital single-lens reflex camera, equipped with a 60 mm lens (Nikon Micro-Nikkor 60mm, Nikon Corporation, Tokyo, Japan) for higher magnification. A mirror lens (Hama angle scope 4399 S7, Hama GmbH & Co KG, Monheim, Germany) allowed the employment of the camera in horizontal directions. Gastropods were animated to feed by custom algae paste (Schneckenfeed Paste, NatureHolic GmbH, Mannheim, Germany) or flour. Small species (e.g. *Trochulus villosulus* or *Stenophysa marmorata*) were filmed with a Keyence VHX-500 digital microscope (KEYENCE, Neu-Isenburg, Germany) and, due to their size, even fed upside down on a Petri dish surrounded by a few water drops (Fig. 3B). Obtained videos (see Movie 1 Supplementary data) were cut and slowed down with Windows Movie Maker 2020 (Microsoft Corporation, Washington, USA) and cropped with Adobe Premiere Pro 2020 (Adobe Inc., San Jose, USA). Schematic illustrations of the radular motion pattern (Figs 4–7) are based on single frames extracted from the videos, which were correlated with scanning electron microscope (SEM) images of the radulae (Figs 4–7 and Supplementary data). For SEM, specimens were dissected, the buccal mass extracted, radulae freed from surrounding tissue and digested with proteinase K according to the protocol of Holznagel (1998), then cleaned for a few seconds in an ultrasonic bath, mounted on an aluminium stub, sputter-coated with carbon and visualised with the SEM Zeiss LEO 1525 (One Zeiss Drive, Thornwood, NY, USA). Images (Figs 4–7 and Supplementary data) were oriented in accordance with the videos and the schematic drawings of the radular motion patterns (Figs 4–7) and not, as in most malacological studies, in traditional way (which would be the other way round except for Heterobranchia). Pictures of shells (Figs 1–2) were taken with a digital camera, Canon EOS 5D Mark III (Canon Inc., Tokyo, Japan) equipped with a Dun stacking system (Dun Inc., Lake Monticello, VA, USA). Photos were then processed with the programs Capture One Pro (Phase One, Copenhagen, Denmark) and Zerene Stacker (Richland, WA, USA) with PMax algorithm. Shells were arranged in a standardised position, in which the aperture is positioned at a 90° angle in relation to the optical axis of the camera and the columella parallel to the background surface. Specimens are inventoried at the Zoological Museum Hamburg (ZMH) of the Centrum für Naturkunde (CeNak), Germany (see Table 1).

## Supplementary Material

Supplementary information

## References

[BIO055699C1] Aktipis S. W., Giribert G., Lindberg D. R. and Ponder W. F. (2008). Gastropoda. An overview and analysis. In *Phylogeny and Evolution of the Mollusca* (ed. Ponder W. F. and Lindberg D. R.), pp. 201-237. California: University of California Press.

[BIO055699C2] Amaudrut M. A. (1898). La partie antérieure du tube digestif de la torsion chez les mollusques gastéropodes. *Ann. Sci. Nat. Zool. Biol. Anim.* 8, 1-291.

[BIO055699C3] Anistratenko V. V., Ryabceva Y. S. and Degtyarenko E. V. (2013). Morphological traits of the radula in Viviparidae (Mollusca, Caenogastropoda) as a master key to discrimination of closely related species. *Vestn. Zool.* 47, 40-51. 10.2478/vzoo-2013-0014

[BIO055699C4] Ankel W. E. (1936). Die Fraßspuren von *Helcion* und *Littorina* und die Funktion der Radula. *Verh. Dtsch. Zool. Ges. Freib.* 38, 174.

[BIO055699C5] Ankel W. E. (1938). Erwerb und Aufnahme der Nahrung bei den Gastropoden. *Verh. Dtsch. Zool. Ges., Zoologischer Anzeiger* 11, 223-295.

[BIO055699C6] Balog G., Voronezhskaya E. E., Hiripi L. and Elekes K. (2012). Organization of the serotonergic innervation of the feeding (buccal) musculature during the maturation of the pond snail *Lymnaea stagnalis*: a morphological and biochemical study. *J. Comp. Neurol.* 520, 315-329. 10.1002/cne.22693 21674495

[BIO055699C7] Carriker M. R. (1943). On the structure and function of the proboscis in the common oyster drill, *Urosalpinx cinerea* Say. *J. Morphol.* 73, 441-506. 10.1002/jmor.1050730304

[BIO055699C8] Carriker M. R., Schaadt J. G. and Peters V. (1974). Analysis by slow-motion picture photography and scanning electron microscopy of radular function in *Urosalpinx cinereal follyensis* (Muricidae, Gastropoda) during shell penetration. *Mar. Biol.* 25, 63-76. 10.1007/BF00395108

[BIO055699C9] Chapman A. D. (2009). *Numbers of Living Species in Australia and the World*, 2nd edn. Toowoomba, Australia: Australian Biodiversity Information Services.

[BIO055699C10] Crampton D. (1977). Functional anatomy of the buccal apparatus of *Onchidoris bilamellata* (Mollusca: Opisthobranchia). *Trans. Zool. Soc. Lond.* 34, 45-86. 10.1111/j.1096-3642.1977.tb00372.x

[BIO055699C11] Crofts D. R. (1929). *Haliotis. Liverpool Marine Biology Committee Memoir 29*. London, UK: Williams and Norgate.

[BIO055699C12] Eigenbrodt H. (1941). Untersuchungen über die Funktion der Radula einiger Schnecken. *Z. Morphol. Oekol. Tiere* 37, 735-791. 10.1007/BF00437763

[BIO055699C13] Evans C. C. E., Dickinson A. J. G. and Croll R. P. (2009). Major muscle systems in the larval Caenogastropod, *Ilyanassa obsoleta*, display different patterns of development. *J. Morphol.* 270, 1219-1231. 10.1002/jmor.10751 19388078

[BIO055699C14] Fretter V. (1965). Functional studies of the anatomy of some neritid prosobranchs. *J. Zool.* 147, 46-74. 10.1111/j.1469-7998.1965.tb01877.x

[BIO055699C15] Fretter V. and Graham A. (1976). *A Functional Anatomy of Invertebrates*. London, UK: Academic Press.

[BIO055699C16] Geddes P. (1879). On the mechanism of the odontophore in certain molluscs. *Trans. Zool. Soc. Lond.* 10, 485-491. 10.1111/j.1096-3642.1879.tb00461.x

[BIO055699C17] Golding R. E., Ponder W. F. and Byrne M. (2009). Three-dimensional reconstruction of the odontophoral cartilages of Caenogastropoda (Mollusca: Gastropoda) using micro-CT: Morphology and phylogenetic significance. *J. Morphol.* 270, 558-587. 10.1002/jmor.10699 19107810

[BIO055699C18] Graham A. (1964). The functional anatomy of the buccal mass of the limpet (*Patella vulgata*). *Proc. Zool. Soc. Lond.* 143, 301-329. 10.1111/j.1469-7998.1964.tb03862.x

[BIO055699C19] Graham A. (1973). The anatomical basis of function in the buccal mass of prosobranch and amphineuran molluscs. *J. Zool. Lond.* 169, 317-348. 10.1111/j.1469-7998.1973.tb04560.x

[BIO055699C20] Gray J. E. (1853). On the division of ctenobranchous gasteropodous Mollusca into larger groups and families. *Ann. Mag. Nat. Hist.* 11, 124-133. 10.1080/03745485609496511

[BIO055699C21] Guralnick R. and Smith K. (1999). Historical and biomechanical analysis of integration and dissociation in molluscan feeding, with special emphasis on the true limpets (Patellogastropoda: Gastropoda). *J. Morphol.* 241, 175-195. 10.1002/(SICI)1097-4687(199908)241:2<175::AID-JMOR7>3.0.CO;2-0 10420163

[BIO055699C22] Haszprunar G. and Götting K. J. (2007). Mollusca, Weichtiere. In *Spezielle Zoologie. Teil 1. Einzeller und wirbellose Tiere* (ed. Westheide W. and Rieger G.), pp. 305-362. Berlin, Germany: Springer.

[BIO055699C23] Haszprunar G., Speimann E., Hawe A. and Heß M. (2011). Interactive 3D anatomy and affinities of the Hyalogyrinidae, basal Heterobranchia (Gastropoda) with a rhipidoglossate radula. *Organ. Divers. Evol.* 11, 201-236. 10.1007/s13127-011-0048-0

[BIO055699C24] Hawkins S. J., Watson D. C., Hill A. S., Harding S. P., Kyriakides M. A., Hutchinson S. and Norton T. A. (1989). A comparison of feeding mechanisms in microphagus, herbivorous, intertidal, *Prosobranchs* in relation to resource partitioning. *J. Moll. Stud.* 55, 151-165. 10.1093/mollus/55.2.151

[BIO055699C25] Herrera S. A., Grunenfelder L., Escobar E., Wang Q., Salinas C., Yaraghi N., Geiger J., Wuhrer R., Zavattieri P. and Kisailus D. (2015). Stylus support structure and function of radular teeth in *Cryptochiton stelleri*. 20th International Conference on Composite Materials Copenhagen, 19-24th July 2015.

[BIO055699C26] Herrick J. C. (1906). Mechanism of the odontophoral apparatus in *Sycotypus canaliculatus*. *Am. Nat.* 40, 707-737. 10.1086/278671

[BIO055699C27] Hickman C. S. (1980). Gastropod radulae and the assessment of form in evolutionary paleontology. *Paleobiology* 6, 276-294. 10.1017/S0094837300006801

[BIO055699C28] Hickman C. S. (1984). Implications of radular tooth-row functional-integration for archaeogastropod systematics. *Malacologia* 25, 143-160.

[BIO055699C29] Hickman C. S. and Morris T. E. (1985). Gastropod feeding tracks as a source of data in analysis of the functional morphology of radulae. *Veliger* 27, 357-365.

[BIO055699C30] Holznagel W. (1998). A nondestructive method for cleaning gastropod radulae from frozen, alcohol-fixed, or dried material. *Am. Malacol. Bull.* 14, 181-183.

[BIO055699C31] Hubendick B. (1956). The eating function in *Lymnaea stagnalis*. *Ark. Zool.* 10, 511-521.

[BIO055699C32] Hyman L. H. (1967). *Mollusca I. Aplacophora, Polyplacophora, Monoplacophora. Gastropoda, the Coelomate Bilateria. The Invertebrates 6*. New York: McGraw-Hill Book Company.

[BIO055699C33] Janssen H. H. and Triebskorn R. (1987). Comparative morphology of the radulae in *Pomatia elegans* and in *Littorina littorea* (Gastropoda: Taenioglossa). *Zool. Anz.* 219, 73-82.

[BIO055699C34] Jörger K. M., Stöger I., Kano Y., Fukuda H., Knebelsberger T. and Schrödl M. (2010). On the origin of *Acochlidia* and other enigmatic euthyneuran gastropods, with implications for the systematics of Heterobranchia. *BMC Evol. Biol.* 10, 323 10.1186/1471-2148-10-323 20973994 PMC3087543

[BIO055699C35] Katsuno S. and Sasaki T. (2008). Comparative histology of radula-supporting structures in Gastropoda. *Malacologia* 50, 13-56. 10.4002/0076-2997-50.1.13

[BIO055699C36] Kehl C. E., Wu J., Lu S., Neustadter D. M., Drushel R. F., Smoldt R. K. and Chiel H. J. (2019). Soft-surface grasping: radular opening in *Alysia californica*. *J. Exp. Biol.* 222, jeb191254 10.1242/jeb.191254 31350299 PMC6739808

[BIO055699C37] Krings W., Faust T., Kovalev A., Neiber M. T., Glaubrecht M. and Gorb S. N. (2019a). In slow motion: radula motion pattern and forces exerted to the substrate in the land snail *Cornu aspersum* (Mollusca, Gastropoda) during feeding. *R. Soc. Open Sci.* 6, 2054-5703. 10.1098/rsos.190222 PMC6689628 31417728

[BIO055699C38] Krings W., Kovalev A., Glaubrecht M. and Gorb S. N. (2019b). Differences in the Young modulus and hardness reflect different functions of teeth within the taenioglossan radula of gastropods. *Zoology* 137, 125713 10.1016/j.zool.2019.125713 31706151

[BIO055699C39] Mackenstedt U. and Märkel K. (1987). Experimental and comparative morphology of radula renewal in pulmonates (Mollusca, Gastropoda). *Zoomorphology* 107, 209-239. 10.1007/BF00312262

[BIO055699C40] Mackenstedt U. and Märkel K. (2001). Radular structure and function. In *The Biology of Terrestrial Molluscs* (ed. Barker G. M.), pp. 213-236. Oxon, UK: CABI Publishing.

[BIO055699C41] Märkel K. (1957). Bau und Funktion der Pulmonaten-Radula. *Zeitschrift für Wissenschaftliche Zoologie* 160, 213-289.

[BIO055699C42] Märkel K. (1964). Modell-Untersuchungen zur Klärung der Arbeitsweise der Gastropodenradula. *Verh. Dtsch. Zool. Ges.* 18, 232-243.

[BIO055699C43] Mikhlina A. L., Tzetlin A. B., Ekimova I. A. and Vortsepneva E. V. (2018). Drilling in the dorid species *Vayssierea* cf. *elegans* (Gastropoda: Nudibranchia): Functional and comparative morphological aspects. *J. Morphol.* 280, 119-132. 10.1002/jmor.20922 30556945

[BIO055699C44] Montroni D., Zhang X., Leonard J., Kaya M., Amemiya C., Falini G. and Rolandi M. (2019). Structural characterization of the buccal mass of *Ariolimax californicus* (Gastropoda; Stylommatophora). *PLoS ONE* 14, e0212249 10.1371/journal.pone.0212249 31390363 PMC6685607

[BIO055699C45] Morris T. E. and Hickman C. S. (1981). A method for artificially protruding gastropod radulae and a new model of radula function. *Veliger* 24, 85-89.

[BIO055699C46] Morse M. P. (1984). Functional adaptations of the digestive system of the carnivorous mollusc *Pleurobranchaea californica* MacFarland, 1966. *J. Morphol.* 180, 253-269. 10.1002/jmor.1051800308 30021399

[BIO055699C47] Neustadter D. M., Drushel R. F. and Chiel H. J. (2002a). Kinematics of the buccal mass during swallowing based on magnetic resonance imaging in intact, behaving *Aplysia californica*. *J. Exp. Biol.* 205, 939-958. 11916990 10.1242/jeb.205.7.939

[BIO055699C48] Neustadter D. M., Drushel R. F., Crago P. E., Adams B. W. and Chiel H. J. (2002b). A kinematic model of swallowing in *Aplysia californica* based on radula/odontophore kinematics and *in vivo* magnetic resonance images. *J. Exp. Biol.* 205, 3177-3206. 12235197 10.1242/jeb.205.20.3177

[BIO055699C49] Padilla D. K. (2003). Form and function of radular teeth of herbivorous molluscs: Focus on the future. *Am. Malacolog. Bull.* 18, 163-168.

[BIO055699C50] Plate L. (1897). Die Anatomie und Phylogenie der Chitonen. *Zool. Jahrb. Suppl. 4 (Fauna chilensis)* 1, 1-227.

[BIO055699C51] Runham N. W. (1962). Rate of replacement of the molluscan radula. *Nature* 194, 992-993. 10.1038/194992b0

[BIO055699C52] Runham N. W. and Thornton P. R. (1967). Mechanical wear of the gastropod radula: a scanning electron microscope study. *J. Zool.* 153, 445-452. 10.1111/j.1469-7998.1967.tb04976.x

[BIO055699C53] Shaw J. A., Macey D. J., Brooker L. R. and Clode P. L. (2010). Tooth use and wear in three iron-biomineralizing mollusc species. *Biol. Bull.* 218, 132-144. 10.1086/BBLv218n2p132 20413790

[BIO055699C54] Simone L. R. L. (2005). Comparative morphological study of representatives of the three families of Stromboidea and the Xenophoroidea (Mollusca, Caenogastropoda), with an assessment of their phylogeny. *Arq. Zool.* 37, 141-267. 10.11606/issn.2176-7793.v37i2p141-267

[BIO055699C55] Simone L. R. L. (2011). Phylogeny of the Caenogastropoda (Mollusca), based on comparative morphology. *Arq. Zool.* 42, 161-323. 10.11606/issn.2176-7793.v42i4p161-323

[BIO055699C56] Solem A. (1972). Malacological applications of scanning electron microscopy II. Radular structure and functioning. *Veliger* 14, 327-336.

[BIO055699C57] Solem A. (1974). *The Shell Makers: Introducing Mollusks*. New York: John Wiley & Sons.

[BIO055699C58] Starmühlner F. (1952). Zur Anatomie, Histologie und Biologie einheimischer Prosobranchier. *Österreichische Zool. Zeit.* 3, 546-590.

[BIO055699C59] Steneck R. S. and Watling L. (1982). Feeding capabilities and limitation of herbivorous molluscs: a functional group approach. *Mar. Biol.* 68, 299-319. 10.1007/BF00409596

[BIO055699C60] Vortsepneva E., Ivanov D., Purschke G. and Tzetlin A. (2013). Morphology of the jaw apparatus in 8 species of Patellogastropoda (Mollusca, Gastropoda) with special reference to *Testudinalia tesulata* (Lottiidae). *Zoomorphology* 132, 359-377. 10.1007/s00435-013-0199-y

[BIO055699C61] Vortsepneva E., Ivanov D., Purschke G. and Tzetlin A. (2014). Fine morphology of the jaw apparatus of *Puncturella noachina* (Fissurellidae, Vetigastropoda). *J. Morphol.* 275, 775-787. 10.1002/jmor.20259 24549973

[BIO055699C62] Wägele H. (1983). Rasterelektronenmikroskopische Untersuchungen an Radulae einiger Nordseeschnecken (Gastropoda: Prosobranchia) mit Anmerkungen zur Funktionsmorphologie. *Drosera* 83, 68-78.

[BIO055699C63] Wanninger A. and Wollesen T. (2019). The evolution of molluscs. *Biol. Rev.* 94, 102-115. 10.1111/brv.12439 PMC6378612 29931833

