# Supplementary material for: Not just scratching the surface: distinct radular motion patterns in Mollusca
Source: Biol Open. 2020 Oct 21;9(10):bio055699. doi: 10.1242/bio.055699 (PMC7595699; doi:10.1242/bio.055699)
Supplement: Supplementary information [file biolopen-9-055699-s1.pdf]

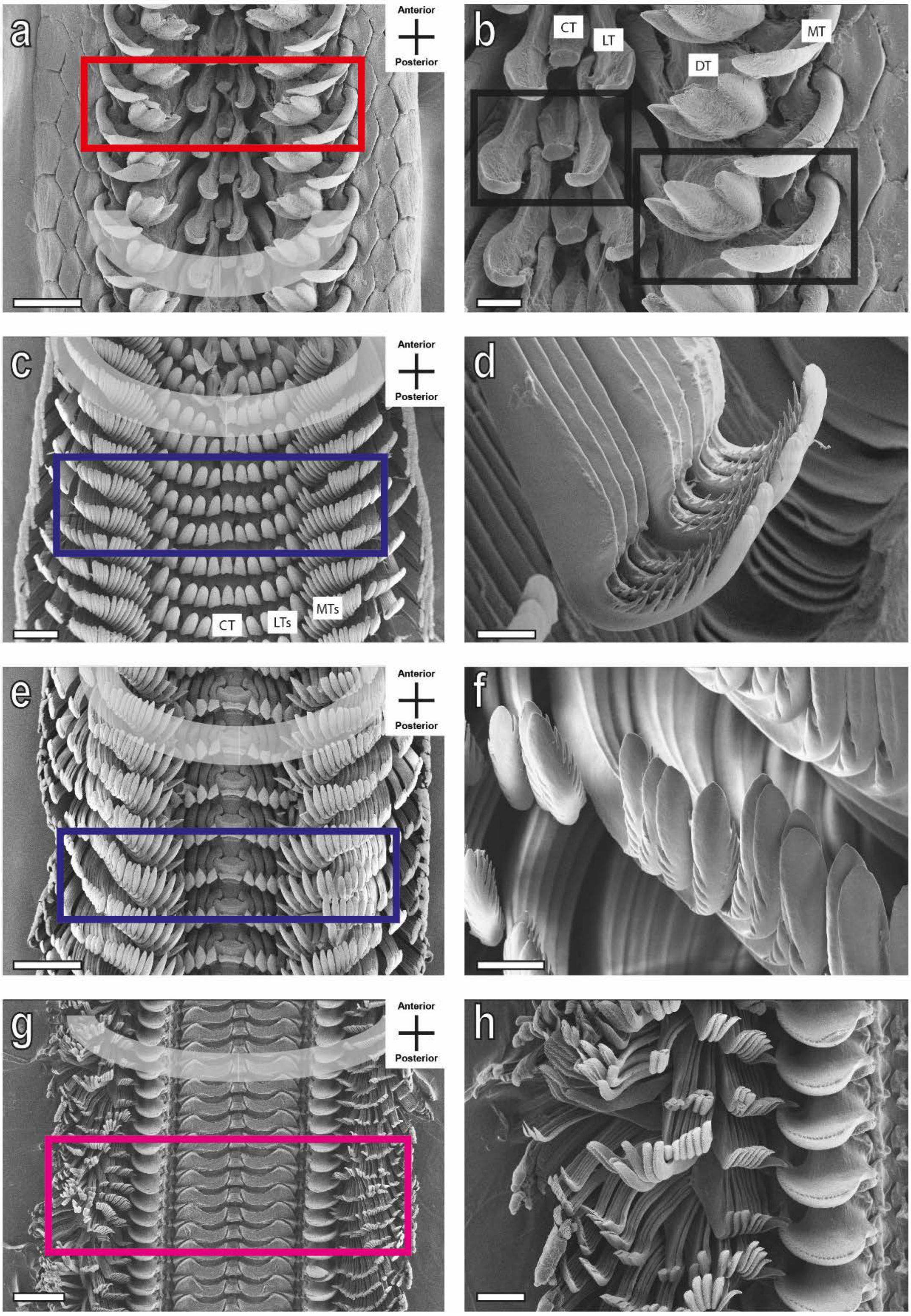

Fig. S1. SEM images of the unworn part of the radula oriented in accordance with video footage, functional rows are highlighted grey, transverse/ontogenetic row is highlighted in black: a-b. *Lepidochitona cinerea*, c-d. *Rochia conus*, e-f. *Astralium calcar*, g-h. *Clithon corona*. Scale bars: a = 100 µm, b, d, f = 30 µm, c = 400 µm, e, g = 200 µm, h = 60 µm. Coloured frames link to Fig. 4-7. CT = central tooth, DT = dominant lateral tooth, LT = lateral tooth, LTs = lateral teeth, MT = marginal tooth, MTs = marginal teeth.

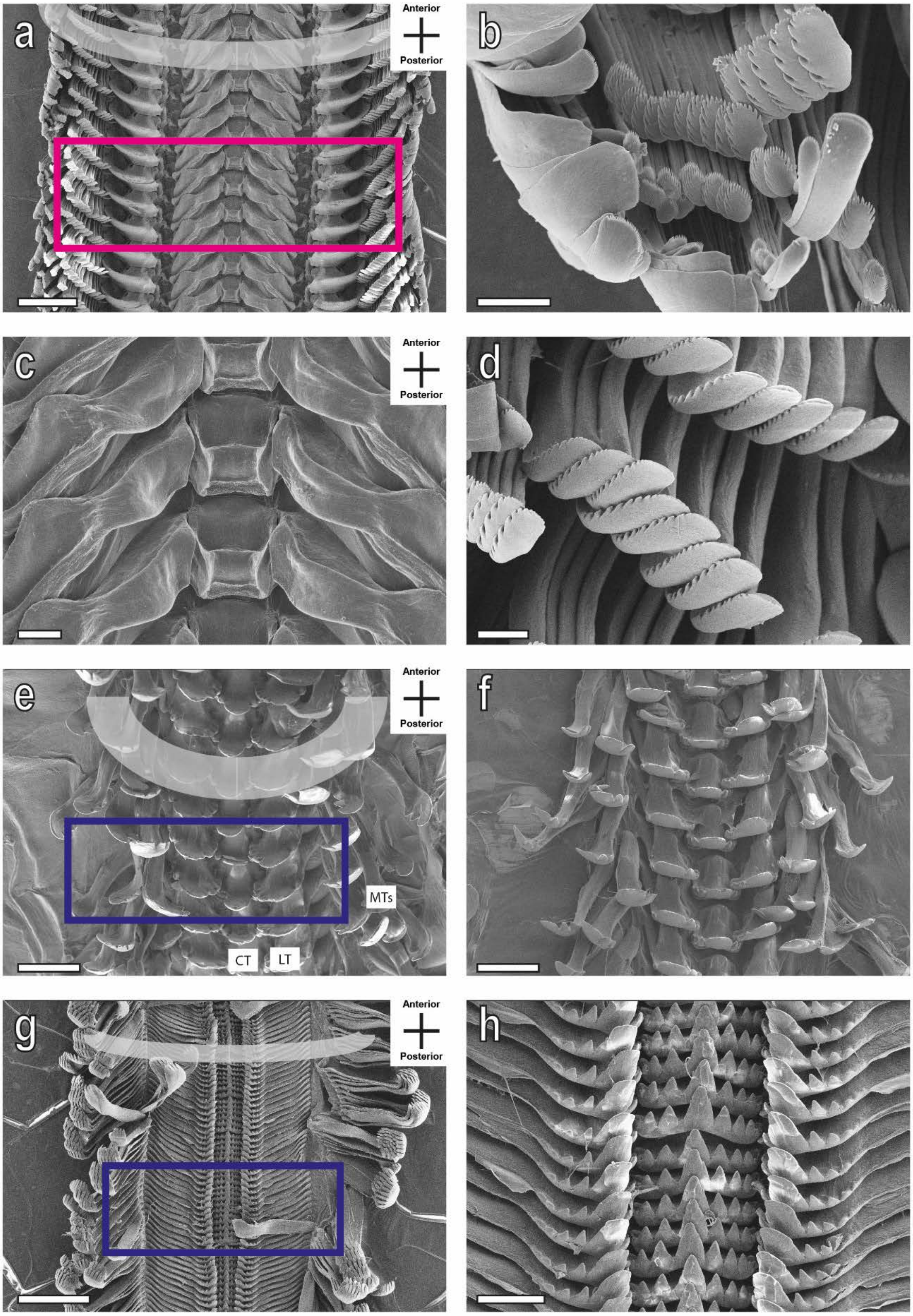

Fig. S2. SEM images of the unworn part of the radula oriented in accordance with video footage, functional rows are highlighted in grey: a-d. *Vittina turrita*, e-f. *Stenomelania torulosa*, g-h. *Thiara cancellata*. Scale bars: a, g = 200 µm, b, d = 20 µm, c, h = 40 µm, e, f = 100 µm. Coloured frames link to Fig. 4-7. CT = central tooth, LT = lateral tooth, MTs = marginal teeth.

Biology Open • Supplementary information

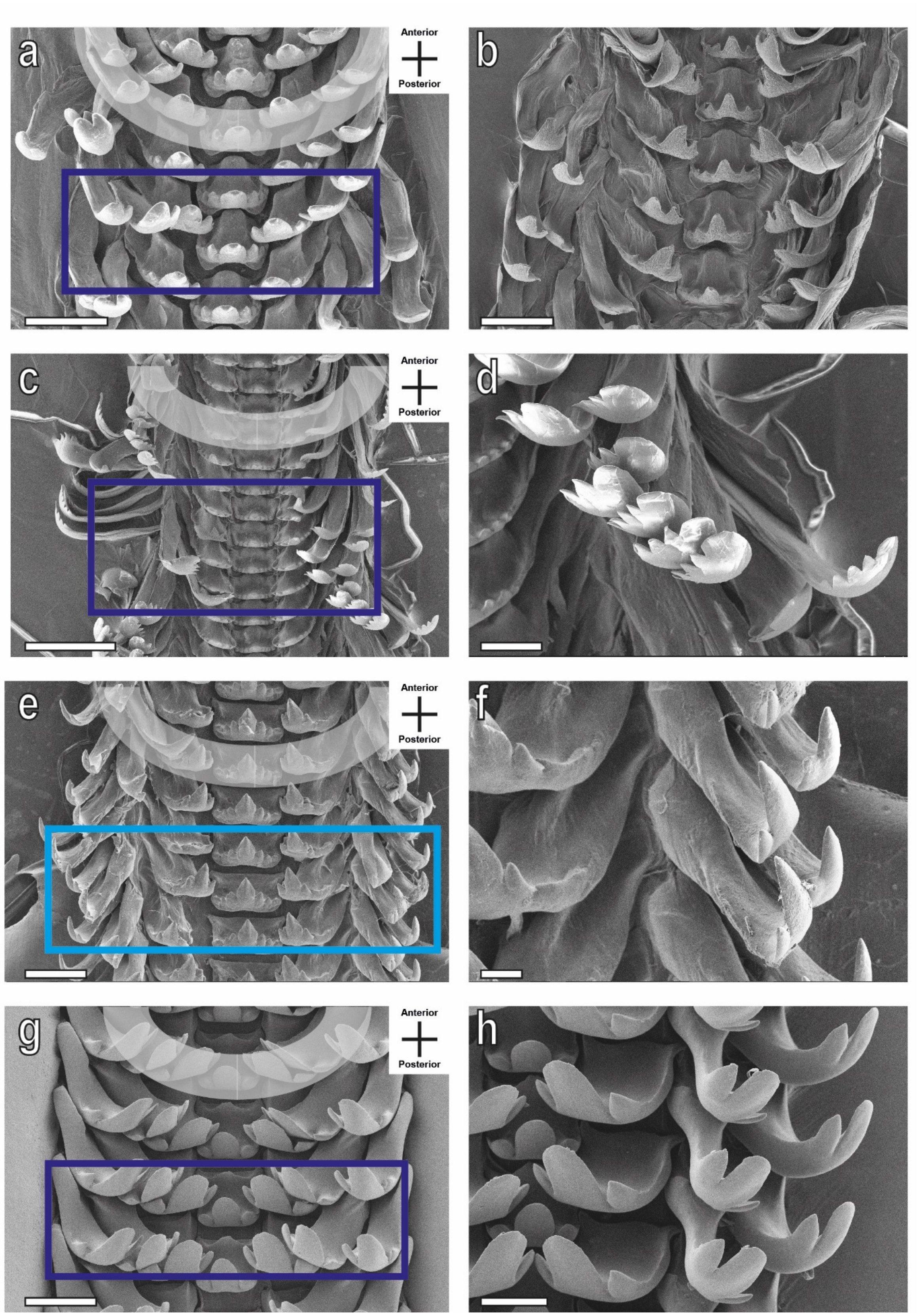

Fig. S3. SEM images of the unworn part of the radula oriented in accordance with video footage, functional rows are highlighted in grey: a-b. *Brotia herculea*, c-d. *Faunus ater*, e-f. *Marisa cornuarietis*, g-h. *Monetaria annulus*. Scale bars: a, b, c, e = 200 µm, d, f, h = 40 µm, g = 100 µm. Coloured frames link to Fig. 4-7.

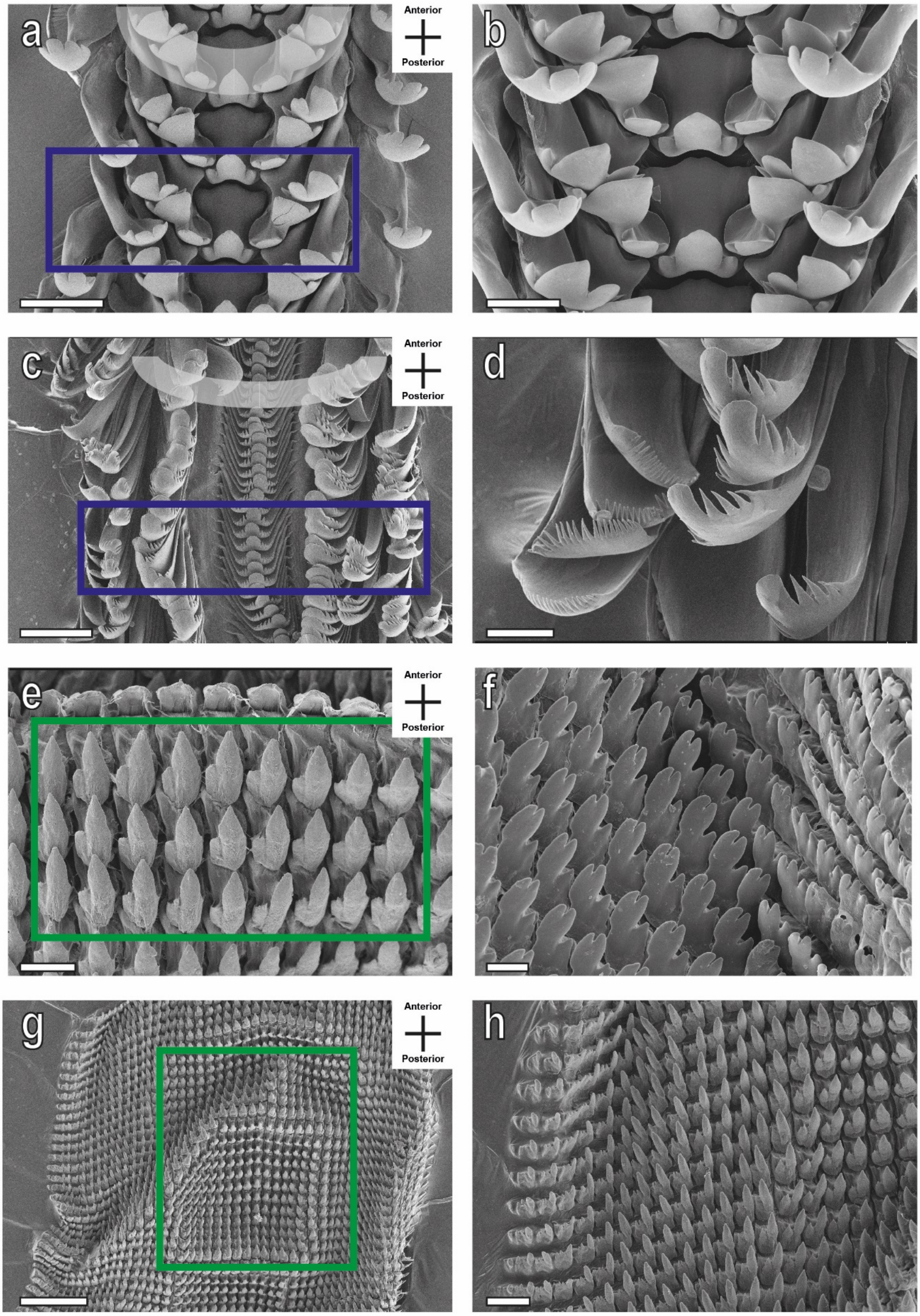

Fig. S4. SEM images of the unworn part of the radula oriented in accordance with video footage, functional rows are highlighted in grey: a-b. *Littorina littorea*, c-d. *Taia naticoides*, e-f. *Cornu aspersum*, g-h. *Trochulus villosulus*. Scale bars: a = 80 µm, b, d, e = 40 µm, c = 200 µm, f, h = 20 µm, g = 100 µm. Coloured frames link to Fig. 4-7.

Biology Open • Supplementary information

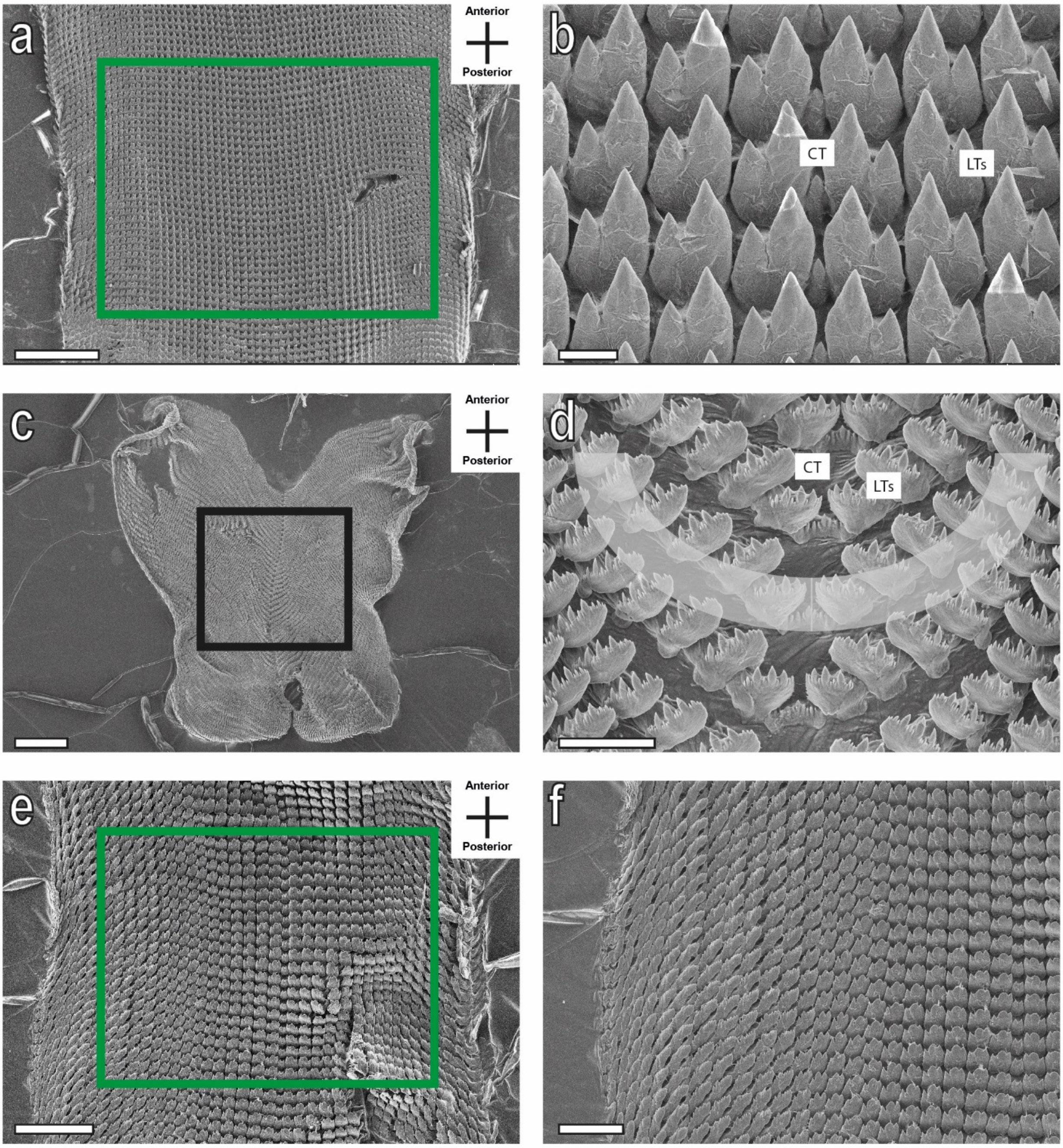

Fig. S5. SEM images of the unworn part of the radula oriented in accordance with video footage, functional rows are highlighted in grey: a-b. *Lymnaea stagnalis*, c-d. *Stenophysa marmorata*, e-f. *Planorbella duryi*. Scale bars: a = 400 µm, b, d = 20 µm, c = 200 µm, e = 100 µm, f = 40 µm. Coloured frames link to Fig. 4-7. CT = central tooth, LTs = lateral teeth.

Biology Open • Supplementary information

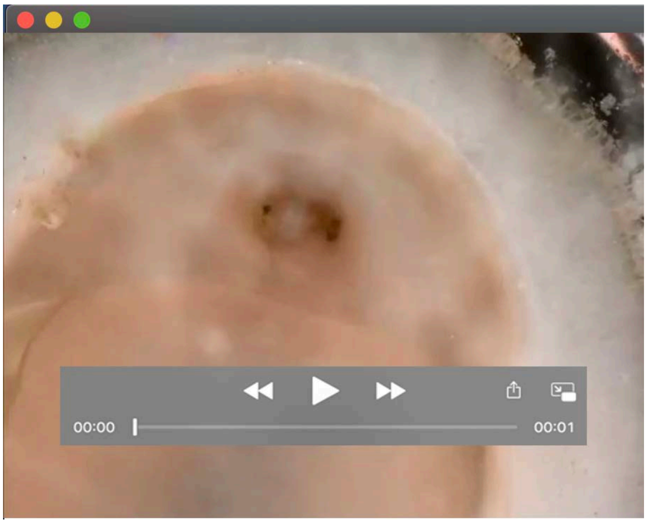

Movie 1

Biology Open • Supplementary information
